# Unchecked nick ligation can promote localized genome re-replication

**DOI:** 10.1016/j.cub.2021.03.043

**Published:** 2021-06-07

**Authors:** Erik Johansson, John F.X. Diffley

**Affiliations:** 1 Chromosome Replication Laboratory, The Francis Crick Institute, London NW1 1AT, UK; 2 Department of Medical Biochemistry and Biophysics, Umeå University, 90187 Umeå, Sweden

## Abstract

Single-stranded DNA breaks, or nicks, are amongst the most common forms of DNA damage in cells. They can be repaired by ligation; however, if a nick occurs just ahead of an approaching replisome, the outcome is a collapsed replication fork comprising a single-ended double-strand break and a ‘hybrid nick’ with parental DNA on one side and nascent DNA on the other (Figure 1A). We realized that in eukaryotic cells, where replication initiates from multiple replication origins, a fork from an adjacent origin can promote localized re-replication if the hybrid nick is ligated. We have modelled this situation with purified proteins *in vitro* and have found that there is, indeed, an additional hazard that eukaryotic replisomes face. We discuss how this problem might be mitigated.

## Teaser

Johansson and Diffley show that a hybrid nick (parental DNA on one side and nascent DNA on the other) at a collapsed fork can be a source of localized re-replication. The results suggest that mechanisms must exist to regulate ligation of the hybrid-nick so that it only happens in conjunction with replication restart to suppress genome instability.

## Main text

**Figure 1:**
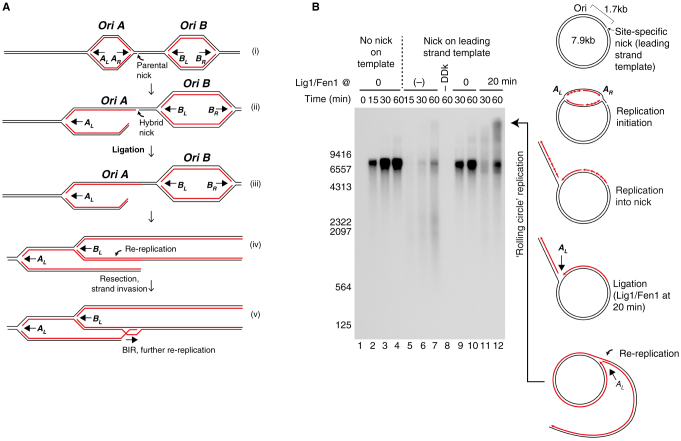
Ligation of a hybrid-nick can promote genome re-replication. (A) A nick on one of the template strands results in a collapsed fork A_R_ and a hybrid nick between a nascent strand (in red) and a parental strand (in black) (i and ii). If the hybrid nick is ligated before fork B_L_ reaches it (iii), then fork B_L_ can continue replicating beyond the position of the nick, resulting in re-replication (red–red dsDNA, iv). Thus, the ligation of the hybrid nick could potentially result in re-replication of large regions of the chromosome. The original broken end can, at any point, be used to initiate break-induced replication (BIR), leading to further re-replication (v). (B) A nick on the leading strand template causes fork A_R_ to collapse. Because of the circularity, fork A_L_ will encounter the collapsed fork. When ligase was added at 20 minutes, high molecular-weight, rolling-circle-replication products were generated by 60 minutes. Taken together, these data show that nascent DNA can be ligated to the template DNA after fork collapse, and can promote localised re-replication.

In *Escherichia coli*, each of the two replication forks from the single chromosomal replication origin (*oriC*) must travel over 2 Mb and are terminated in a zone ∼180° from the origin (Figure S1A). Consequently, the only way to complete replication after fork collapse is to restart the collapsed fork^1^^,^^2^. In replication restart — known as ‘break-induced replication’ in eukaryotes — the hybrid nick is ligated, the broken end is resected by a 5’->3’ nuclease, and a recombinase promotes strand invasion of the single stranded 3’ overhang (Figure S1B)^1^^,^^3^. A new replisome can then assemble at this primer to complete replication. In eukaryotes, the genome is replicated from multiple origins and individual replication forks travel much shorter distances than in *E. coli*. There are many ‘dormant’ replication origins that can be activated if needed and, outside ribosomal DNA, there are no programmed termination zones^4^. In contrast to *E. coli*, therefore, a collapsed replication fork (for example, fork A_R_ in Figure 1A, diagrams i and ii) will likely be met by a converging replication fork from a downstream origin (fork B_L_ in Figure 1A). The fate of the hybrid nick then determines the outcome of this encounter. If the hybrid nick remains unligated (Figure S1C), fork B_L_ will also collapse, generating the equivalent of a ‘clean’ double strand break, which can be repaired by non-homologous end joining or homologous recombination using the sister chromatid as a template^3^. If, however, the hybrid nick is ligated before fork B_L_ reaches it (Figure 1A, diagram iii) — and regardless of whether the original nick was on the leading or lagging strand template — fork B_L_ can continue replicating beyond the position of the nick, now using the previously replicated DNA as template leading to localized re-replication (red–red dsDNA in Figure 1A, diagram iv). This rogue replication fork will now be following the partner of the original collapsed fork (Fork A_L_ in Figure 1A, diagram iv), potentially re-replicating large regions of the chromosome. The original broken end can, at any point, be used to initiate break-induced replication, leading to further re-replication (Figure 1A, diagram v).

To assess the plausibility of these ideas, we needed to determine firstly whether hybrid nicks generated during eukaryotic DNA replication *in vitro*^5^^,^^6^ can be ligated (Figure S2) and secondly whether ligation could promote re-replication (Figure 1B). To assess the ligation of the hybrid nick, we introduced a sequence 1.7 kb away from the origin that can be nicked on the leading or lagging strands by the Nb.BbvCI and Nt.BbvCI restriction nucleases, respectively. We then asked whether nascent DNA can be ligated efficiently to template DNA at a nick on the lagging-strand template as outlined in Figure S2A. A novel product (labeled ‘product 1’), larger than full-length plasmid, was generated when ligase was added at 20 or 40 minutes after initiation (Figure S2A). This product is the size (∼ 9.4 kb) predicted if the hybrid nick was ligated.

As a second approach to examine ligation of the hybrid nick, we replicated a linearised plasmid containing a nick on the lagging strand (Figure S2B,C) and digested the replicated products with the Dpn1 restriction enzyme. Dpn1 does not digest hemi-methylated DNA, but efficiently digests input plasmid DNA, which is fully methylated in *E. coli*. When ligase was added to the nicked template 20 or 40 minutes after initiation, the full-length products (Figure S2C, lanes 14–17) were lost after DpnI digestion (Figure S2C, lanes 23–26), and a novel band of ∼ 2.4 kb was generated. Together, these experiments show that hybrid nicks can be ligated efficiently *in vitro*.

We next sought to determine if replication into ligated hybrid nicks could generate re-replication. To test this notion, we used a circular plasmid as template (Figure 1B) with the complete replication system including the Okazaki fragment synthesis and maturation machinery (Pol δ, RFC, PCNA and Fen1). The plasmid was nicked on the leading strand template causing fork A_R_ to collapse; ligase was added at either t = 0 or t = 20 minutes. Because of the plasmid’s circularity (Figure S2D), fork A_L_ (rather than Fork B_L_) will encounter the collapsed fork: if fork A_L_ can replicate past the hybrid nick, it will generate ‘rolling-circle replication’ products (Figure 1B). When ligase was added at 20 minutes, in addition to full-length products, high molecular weight rolling-circle replication products were generated by 60 minutes (Figure 1B, lane 12). Taken together, these data show that nascent DNA can be ligated to the template DNA after fork collapse, and can promote localised re-replication.

Our results indicate that a collapsed fork can be a source of localized re-replication. We suggest that mechanisms must exist to regulate ligation of this nick so that it only happens in conjunction with replication restart. Perhaps poly-ADP-ribose polymerase, which binds tightly to nicks and recruits repair factors, plays a role in this regulation^7^. Poly-ADP-ribose polymerase is absent from yeast, so other mechanisms may also exist. Nicks can come from multiple sources including incomplete DNA repair and topoisomerase reactions. Hybrid nicks can also be generated by cleavage of stalled replication forks by structure-specific nucleases including Mus81^8^. Consequently, mechanisms involved in regulating this ligation event are likely to be important for genome stability.

## Supplemental information

Document S1. Two supplemental figures, experimental procedures, author contributions and supplemental references
