# Supplementary material for: Unchecked nick ligation can promote localized genome re-replication
Source: Curr Biol. 2021 Jun 7;31(11):R710–1. doi: 10.1016/j.cub.2021.03.043 (PMC8209288; doi:10.1016/j.cub.2021.03.043)
Supplement: Document S1. Two supplemental figures, experimental procedures, author contributions and supplemental references [file mmc1.pdf]

Supplemental Information:

Unchecked nick ligation can promote localized genome re-replication

Erik Johansson and John F.X. Diffley

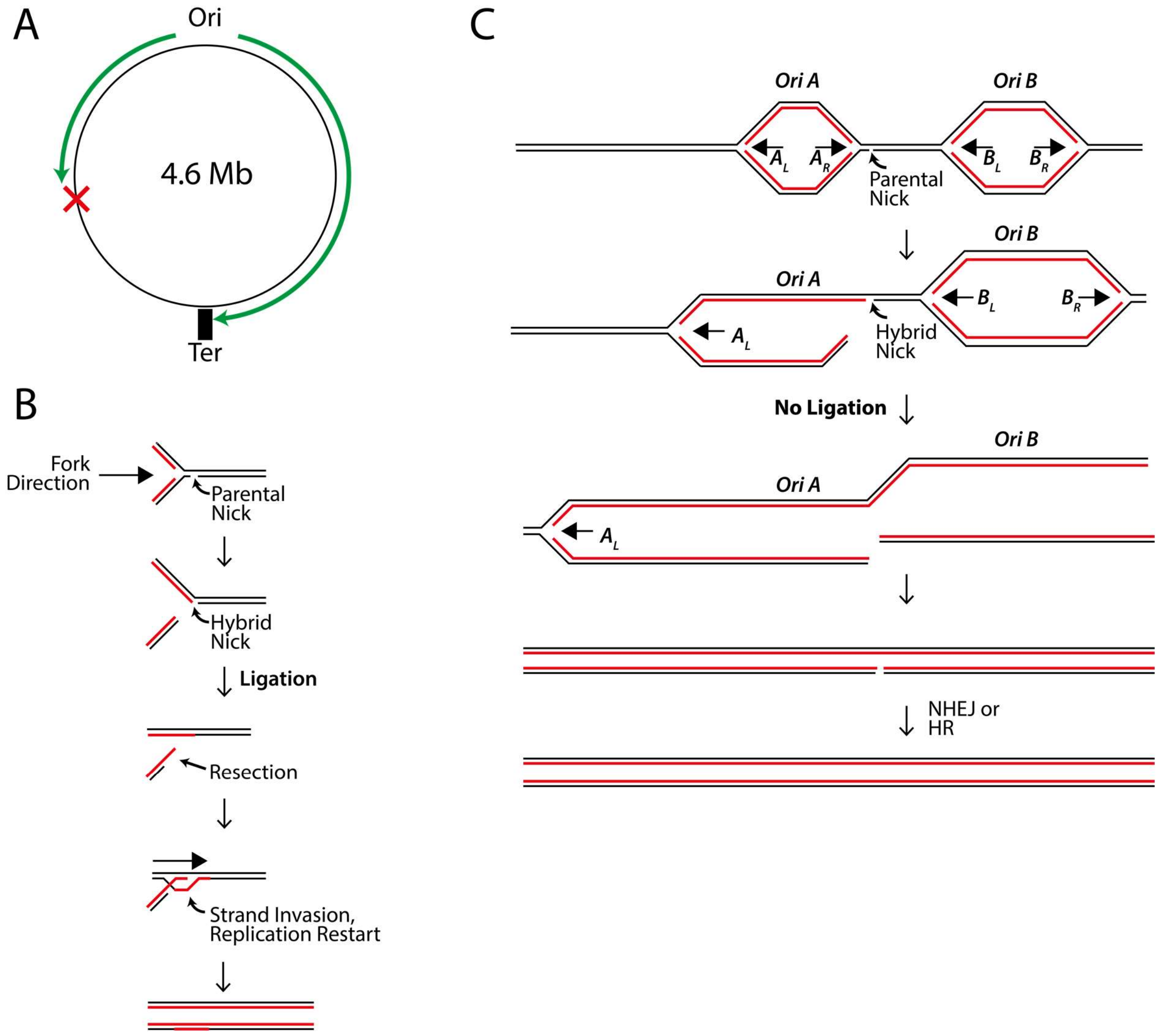

Figure S1: Schemes illustrating different scenarios following a nick-induced collapse of the replication fork.

A) The *Escherichia coli* genome is circular with one origin and a replication termination zone located 180° from the origin. When a replication fork collapses, it must be restarted since the other fork cannot proceed through the termination zone. B) Replication restart mechanism. A nick on one strand results in a collapsed fork with a one-sided double-strand break and a hybrid nick. The hybrid nick is ligated and 5´->3´resection creates a stretch of single-stranded DNA. The single-stranded DNA invades the duplex DNA, base-pairing with a complementary sequence. A 'D-loop' is formed and DNA replication is resumed. C) A nick on one of the template strands results in a collapsed fork A_R and a hybrid nick between a nascent strand (in red) and a parental strand (in black). If unligated, the B_L fork also

collapses at the hybrid nick and a conventional double-strand break is created that can be repaired by non-homologous end-joining (NHEJ) or homologous recombination (HR).

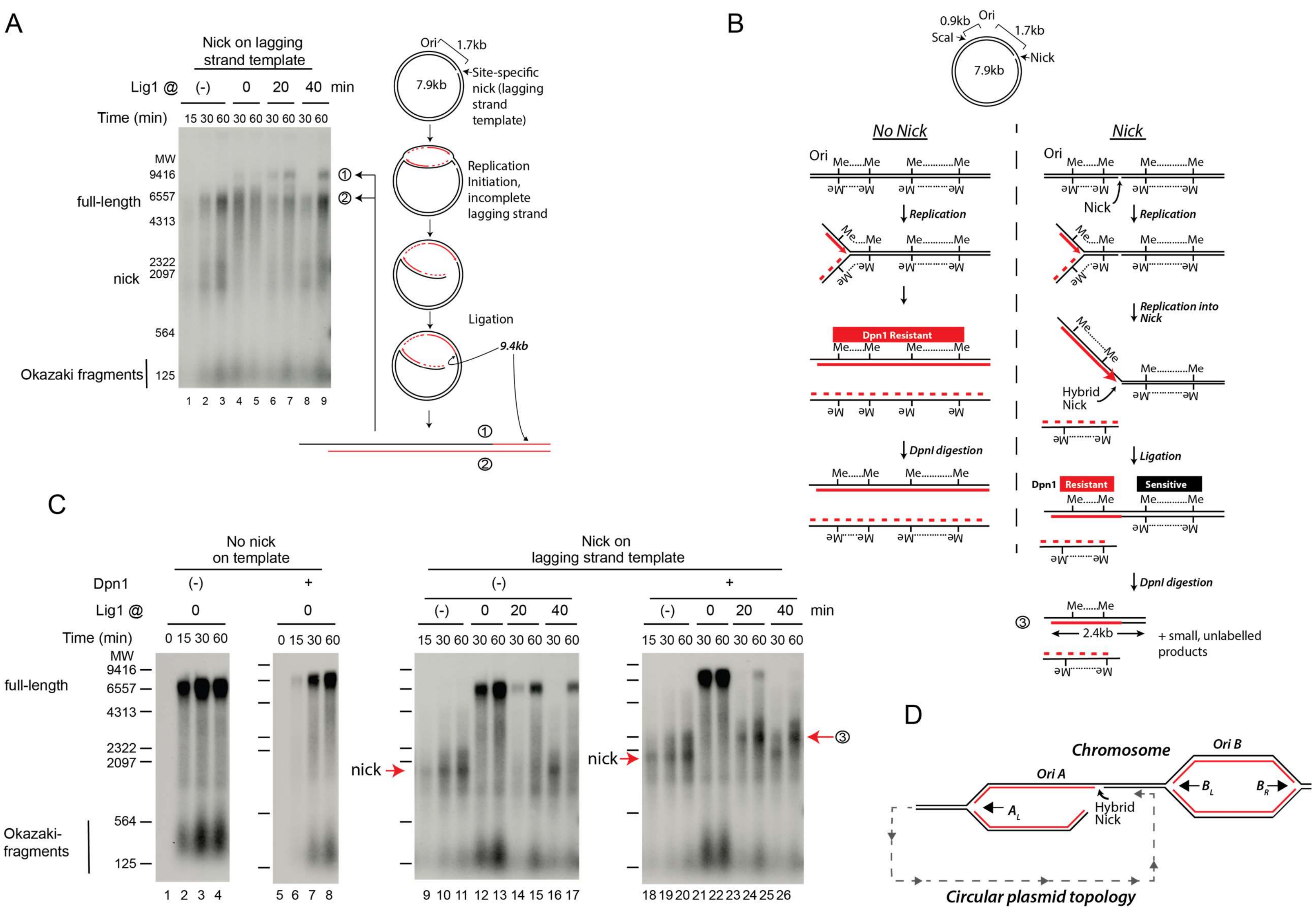

A
Nick on lagging strand template
Lig1 @ (-) 0 20 40 min
Time (min) 15 30 60 30 60 30 60 30 60
MW
9416
6557
4313
2322
2097
564
125
full-length
nick
Okazaki fragments
1 2 3 4 5 6 7 8 9
①
②
Ori
1.7kb
7.9kb
Site-specific nick (lagging strand template)
Replication Initiation, incomplete lagging strand
Ligation
9.4kb
B
0.9kb
Ori
1.7kb
ScaI
Nick
7.9kb
No Nick
Nick
Ori
Me......Me
Me............Me
Replication
Replication into Nick
Hybrid Nick
Dpn1 Resistant
Ligation
Dpn1
Resistant
Sensitive
DpnI digestion
③
2.4kb
+ small, unlabelled products
C
No nick on template
Nick on lagging strand template
Dpn1 (-) + (-) +
Lig1 @ 0 0 (-) 0 20 40 (-) 0 20 40 min
Time (min) 0 15 30 60 0 15 30 60 15 30 60 30 60 30 60 30 60 15 30 60 30 60 30 60 30 60
MW
9416
6557
4313
2322
2097
564
125
full-length
Okazaki-fragments
nick
③
1 2 3 4 5 6 7 8 9 10 11 12 13 14 15 16 17 18 19 20 21 22 23 24 25 26
D
Chromosome
Ori A
Ori B
Hybrid Nick
Circular plasmid topology

Figure S2: Probing whether a hybrid nick at a collapsed replication fork can be ligated.

To prevent longer Okazaki fragments from confusing the analysis, reactions did not contain DNA polymerase δ (Pol δ), RFC or PCNA; thus Okazaki fragments can initiate (because pol α is present), but cannot be extended into full-length Okazaki fragments. (A) Probing whether nascent DNA is ligated to template DNA on a circular substrate when the first fork runs into a nick on the lagging strand template. Ligase 1 was added 20 or 40 minutes after the initiation of DNA replication, giving the fork time to reach the nick before ligase was added. The longest product (1) is the result of a ligation event between the nascent DNA and template DNA. (B) The scheme illustrates possible outcomes when DpnI is incubated with replication products. DpnI cuts DNA purified from *E. coli* but not in vitro-replicated, hemi-methylated DNA. Completely replicated products digested with DpnI should generate a full-length DNA strand in alkaline agarose gels, and this is what is seen when either unnicked template is replicated or ligase is added at time t=0 (please compare lanes 1–4 with 5–8 and 12,13 with 21,22 in C). If the hybrid nick is ligated, the full-length labelled product should be part DpnI-sensitive and part DpnI-resistant. (C) Probing whether nascent DNA is ligated to template DNA on a linearized substrate when the fork encounters a nick on the lagging strand template. Ligase 1 was added 20 or 40 minutes after the initiation of DNA replication, giving the fork time to reach the nick before ligase was added. Replication products in lanes 5–8 and 18–26 were incubated with DpnI. The length of products terminating at a nick are shown. A 2.4 kb fragment (labelled 3) was observed when the hybrid nick at the collapsed fork was ligated and the products were digested by DpnI (please see 3 in B). D) Fork A_R (Figure 1A on chromosome and Figure 1B on circular plasmid) has collapsed at a nick. Fork A_L in Figure 1B will proceed around the circular plasmid and encounter the hybrid nick from the same direction as fork B_L does on a linear chromosome (Figure 1A). Thus, a single origin (OriA) on a circular plasmid can model events that occur between two separate origins (OriA and OriB) on a linear chromosome.

Supplemental experimental procedures

Materials availability:

Requests for reagents and strains should be directed to John F.X. Diffley.

Over-expression of recombinant proteins

Cdc6, GINS, Mcm10, PCNA were over-expressed in bacteria as described previously[S1-S3]. BL21(DE3)-CodonPlus-RIL cells carrying the Sld2 over-expression plasmid were grown in LB with 100 µg/ml ampicillin and 37 µg/ml chloramphenicol at 37° C. At OD_{600nm}=0.5, cells were cooled to 16° C and 0.2 mM IPTG was added to induce over-night expression. ORC, Mcm2-7/Cdt1, DDK, S-CDK, Cdc45, Dpb11, Sld3/Sld7, Pol ε, RPA, Pol α, Ctf4, MrcI, Csm3/Tof1, TopoI, RFC, Pol δ, Fen1, Lig1 were over-expressed in yeast as previously described[S1-S5].

Purification of proteins

A detailed protocol of how ORC, Mcm2-7/Cdt1, DDK, S-CDK, Cdc45, Dpb11, Sld3/Sld7, Pol ε, RPA, Pol α, Ctf4, MrcI, Csm3/Tof1, TopoI, RFC, Pol δ, Fen1, Lig1, Cdc6, GINS, Mcm10, and PCNA were purified will be described elsewhere. The proteins were purified essentially as previously described[S1-S5] with minor changes.

Sld2 was over-expressed from plasmid pGC441 (a kind gift from Gideon Coster) in BL21(DE3)-CodonPlus-RIL cells. The lysate was incubated with Glutathione Sepharose in 25 mM HEPES-KOH (pH 7.6), 10% glycerol, 500 mM NaCl, 1 mM EDTA, 0.02% NP-40-S, 0.1% Tween20, 1 mM dithiothreitol, and the recommended concentration of a protease cocktail from cOmplete Mini, EDTA-free (Roche). After extensive washing, Sld2 was released from the affinity resin by incubation with PreScission protease. The eluate was pooled and diluted with an equal volume of buffer with no salt to reduce the salt concentration to 250 mM NaCl, and thereafter loaded onto a 1 mM HiTrap SP FF column equilibrated in 25 mM HEPES-

KOH (pH 7.6), 10% glycerol, 250 mM NaCl, 1 mM EDTA, 0.02% NP-40-S, 0.1% Tween20, and 1 mM dithiothreitol. The peak fractions from a 10 CV step elution in the same buffer with 700 mM NaCl were pooled and dialyzed against 25 mM HEPES-KOH (pH 7.6), 40% glycerol, 700 mM KOAc, 1 mM EDTA, 0.02% NP-40-S, and 1 mM dithiothreitol.

The template for the in vitro replication assay

The plasmid used as a template, CEJ5, in the in vitro replication assay was derived from plasmid MD154 (a kind gift from Max Douglas). A linker with a unique recognition site for Nt.BbvCI and Nb.BbvCI was introduced about 1.7 kb from the eukaryotic origin of replication. Nt.BbvCI or Nb.BbvCI (New England Biolabs) was used to create a single nick in the plasmid, positioned on either the leading or lagging strand template for an approaching replication fork. A unique restriction site, ScaI on the opposite side of the origin allowed the plasmid to be linearized when indicated.

Replication assays

In vitro replication assays were performed as previously described[S2,S3] but with the following modifications. MCM loading was carried out by incubating either circular or linearized 4 nM plasmid DNA (CEJ5) with 8 nM ORC, 15 nM Cdc6, 22.5 nM Mcm2-7/Cdt1, 5 mM ATP, 25 mM HEPES-NaOH (pH 7.6), 100 mM potassium glutamate, 10 mM $Mg(Oac)_2$, 2 mM dithiothreitol, and 0.02% NP-40-S at 30° C for 10 minutes. Next, the reaction was supplemented with 20 nM Dbf4-dependent kinase (DDK) and incubated for an additional 5 minutes at 30° C. The loading reaction was diluted two-fold upon the initiation of DNA replication when the following reagents were added (final concentrations): 5 nM S-CDK, 7.5 nM Dpb11, 15 nM GINS, 120 nM Cdc45, 15 nM Pol ε, 7.5 nM Mcm10, 5 nM Ctf4, 50 nM RPA, 15 nM Csm3/Tof1, 20 nM Mrc1, 5 nM TopoI, 80 nM Pol α, 7.5 nM Sld3/Sld7, 9 nM Sld2, 200 μM CTP, 200 μM GTP, 200 μM UTP, 80 μM dATP, 80 μM dGTP, 80 μM dTTP, 80 μM dCTP and 1 μCi [α-^{32}P]-dCTP. The final buffer

condition, including salt and glycerol supplemented by the added proteins was: 30 mM HEPES-NaOH (pH 7.6), 16 mM $Mg(Oac)_2$, 3 mM dithiothreitol, 0.02% NP-40-S, 11 mM KCl, 48 mM KAc, 250 mM potassium glutamate, and <2% glycerol. 20 nM ligase (Cdc9) was added as indicated and in addition 20 nM RFC, 100 nM PCNA, 5 nM Pol δ, 20 nM Fen1 and increased the final KCl concentration to 60 mM when processing of Okazaki-fragments was required. Reactions were incubated at 30 °C for up to 60 minutes after which they were quenched by the addition of 65 mM EDTA before removal of unincorporated nucleotides using Illustra G-50 columns (GE Healthcare). The products were separated on a 1% alkaline agarose gel, run for 16 hours at 30 V as described earlier[S3]. The alkaline agarose gels were fixed by two sequential 20-minute incubations in 5% trichloroacetic acid solution at room temperature before drying on 3MM chromatography paper. Gels were autoradiographed using Amersham Hyperfilm MP (GE Healthcare).

Author Contributions

E.J. executed the original experiments, E.J. and J.F.X.D. conceived the experiments, analysed the data, and wrote the paper.

Supplemental references

S1 Frigola, J., Remus, D., Mehanna, A., and Diffley, J.F. (2013). ATPase-dependent quality control of DNA replication origin licensing. Nature *495*, 339–343.

S2 Yeeles, J.T., Deegan, T.D., Janska, A., Early, A., and Diffley, J.F. (2015). Regulated eukaryotic DNA replication origin firing with purified proteins. Nature *519*, 431–435.

S3 Yeeles, J.T.P., Janska, A., Early, A., and Diffley, J.F.X. (2017). How the eukaryotic replisome achieves rapid and efficient DNA replication. Mol. Cell *65*, 105–116.

S4 On, K.F., Beuron, F., Frith, D., Snijders, A.P., Morris, E.P., and Diffley, J.F. (2014). Prereplicative complexes assembled in vitro support origin-dependent and independent DNA replication. EMBO J. *33*, 605–620.

S5 Coster, G., Frigola, J., Beuron, F., Morris, E.P., and Diffley, J.F. (2014). Origin licensing requires ATP binding and hydrolysis by the MCM replicative helicase. Mol. Cell *55*, 666–677.
